# Sex-Linked Molecular Markers Identify Female Lines in Endosperm-Derived Kiwifruit Callus and in Regenerants

**DOI:** 10.3390/plants10030526

**Published:** 2021-03-11

**Authors:** Iwona Chłosta, Dagmara Kwolek, Elwira Sliwinska, Grzegorz Góralski, Marzena Popielarska-Konieczna

**Affiliations:** 1 Department of Plant Cytology and Embryology, Institute of Botany, Faculty of Biology, Jagiellonian University in Kraków, Gronostajowa 3, 30-387 Kraków, Poland; Iwona.kleszcz@doctoral.uj.edu.pl (I.C.); dagmara.kwolek@uj.edu.pl (D.K.); g.goralski@uj.edu.pl (G.G.); 2 Department of Agricultural Biotechnology, Faculty of Agriculture and Biotechnology, UTP University of Science and Technology in Bydgoszcz, Kaliskiego 7, 85-789 Bydgoszcz, Poland; elwira@utp.edu.pl

**Keywords:** *Actinidia chinensis* var. *deliciosa*, endosperm culture, flow cytometry, hexaploids, nonaploids

## Abstract

This is the first report of molecular markers application for the analysis of endosperm-derived callus and nonaploid kiwifruit (*Actinidia chinensis* var. *deliciosa,* formerly: *Actinidia deliciosa*) plants. As a source of explants, fruits of ‘Hayward’, the most popular cultivar, were used. Additionally, analyses of the nuclear DNA content and sex were conducted on the regenerated plants. Hexaploid seedlings were used as control for the flow cytometric analyses. Most of the plants (about 90%) regenerated via endosperm-derived callus possessed 2C = 9Cx DNA, which confirmed their endosperm origin and nonaploidy. Because *Actinidia* is a dioecious species, and female plants bearing fruits are desired by breeders, it is crucial to identify the sex of an individual at early stages of development. Analyses were conducted with ex vitro and in vitro samples. Results revealed that specific markers for a Y-chromosome applied at the callus stage allowed us to reliably predict the sex of plants regenerated from it. This is a novel application of sex-linked markers for early selection of female and male callus lines when the sex of the initial explants is still unknown, such as fresh isolated embryos and endosperm. It may have significant importance for breeding kiwifruit programs, which involve tissue culture techniques.

## 1. Introduction

Polyploidization, a common phenomenon among plants, plays an important role in the speciation mechanism in a natural environment as well as in breeding programs in agriculture [1,2]. One of the techniques used to induce polyploidy is to start with an in vitro culture of endosperm tissue [3,4]. This storage tissue has a higher ploidy than the embryo in the majority of Angiosperms. Additionally, it is possible to induce polyploid plants with 3n number of chromosomes by plant regeneration from endosperm-derived callus cultured in vitro, and bypassing the genomic conflict in endosperm [5]. In vitro-produced plants with a higher ploidy status may display unique features such as larger cells (including epidermal and/or guard cells) and organs (e.g., leaves, flowers, fruits) [2]. Plants of the genus *Actinidia* with a basic number of chromosomes x = 29 includes 75 taxa with 54 species and 21 varieties; these taxa include the most frequently planted vine trees in the world [6]. The most popular and commercially grown species is *A*. *chinensis* var. *deliciosa* A. Chev. (A. Chev.), formerly known as *A*. *deliciosa* (A. Chev.) C.F. Liang et A.R. Ferguson, commonly called kiwifruit [7]. *A*. *chinensis* var. *deliciosa* belongs to the *A*. *chinensis* complex, populations of which show different combinations of di-, tetra-, penta-, and hexaploids [8]. Efforts have been made to obtain kiwifruit plants with different ploidy levels, such as triploids (after pollination with lethally-irradiated pollen [9]) and dodecaploids (during multiplication and unprompted polyploidization [10]). Another method to increase ploidy is the production of interspecific hybrids using crosses between species with different ploidies [11]. Successful attempts at hybridization between *Actinidia* species have been made using cross-pollination [12] combined with in vitro embryo-rescue [13]. Although plant regeneration from endosperm was reported for diploid cultivar of *A. chinensis* [14], for hexaploid kiwifruit *A. chinensis* var. *deliciosa* [6,7] ‘Hayward’, only organogenesis and shoot buds formation from endosperm-derived callus were reported [15].

The kiwifruit is a dioecious species; the sex is chromosomally determined: (X)_4_ XX in the females and (X)_4_ XY in the males [16,17]. A heterogametic male system with homomorphic sex chromosomes regulates the development of the gynoecium (female flower) and androecium (male flower). In the *Actinidia* genus, the Y chromosome determines the maleness of a plant, regardless of the number of X chromosomes and ploidy [18]. Numerous studies to determine the sex-determinant region have been mainly focused on economically important species, especially *A*. *chinensis* var. *chinensis* and *A*. *chinensis* var. *deliciosa* [19]. Proposals for presumed X- or Y-specific sequences in the genome suggest that some autosomal genetic factors can influence sex determination [20,21,22]. Recently, the cytokinin-signaling gene *Shy Girl* (*SyGl*), which is located on the Y chromosome, was found to suppress the development of the female flower organs [23], which resulted in male flower development; this is the case for the entire *Actinidia* genus. A second proposed gene called *Friendly Boy* (*FrBy*) is also located on the Y chromosome. It acts independently of *SyGl* and seems to be present in other dioecious species [23], and its expression in female kiwifruit plants results in their hermaphroditism [24]. Dioecy and an extended juvenile phase, as many as four to eight years for kiwifruit [25], is an issue, because identifying fruit-bearing females at an early stage of the growth is crucial for breeders [26]. For this reason, in addition to morphological and biochemical markers, molecular markers are very useful to identify female and male plants at an early stage of development.

The main goal of the work was to develop a method for the effective selection of the desired female culture lines from endosperm-derived callus and regenerated plants by the use of sex-linked molecular markers, which might be valuable for kiwifruit breeding. To achieve this goal we have (1) regenerated and acclimatized plants to ex vitro conditions, (2) performed flow cytometric analyses of control seedlings and regenerated plants to establish nuclear DNA content and ploidy, and (3) tested the molecular sex-linked markers to determine the sex of the callus and plants regenerated from endosperm-derived callus.

## 2. Results

### 2.1. Callus Lines, Plant Regeneration, and Acclimatization

**Figure 1:**
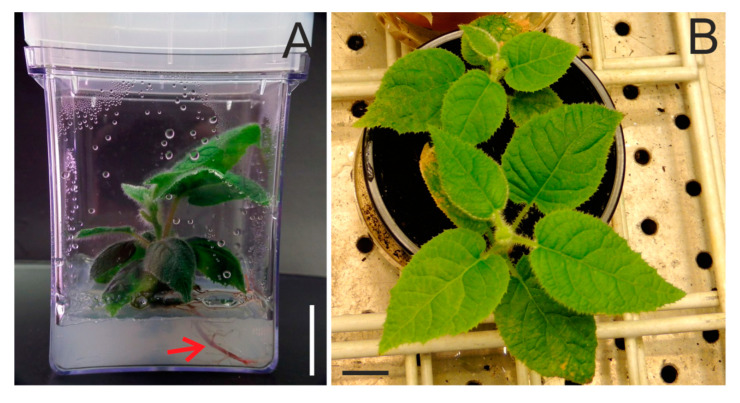
Rooting and acclimatization of the *Actinidia chinensis* var. *deliciosa* plants to ex vitro conditions. (**A**) Plants regenerated from endosperm-derived callus and transferred onto half-strength MS medium in Magenta vessels; visible well-developed roots (arrow). (**B**) Acclimated plants. Bars = 2 cm.

Twenty one endosperm-derived callus lines were obtained and multiplied for over four years. Each callus line produced from one to eighteen shoot buds (in total: 136, average: 6.4 shoots per callus line). The shoot buds increased their size to 1–2 cm, and then were transferred onto half-strength MS medium to induce the development of the roots (Figure 1A). The efficiency of rooting achieved was 90.4% (in total: 123 rooted shoot buds). Callus formation at the basal end of the stem was observed sporadically. After the transfer to pots, 78.9% of rooted plants (97) were successfully acclimated (Figure 1B).

### 2.2. Nuclear DNA Content and Ploidy Range

**Figure 2:**
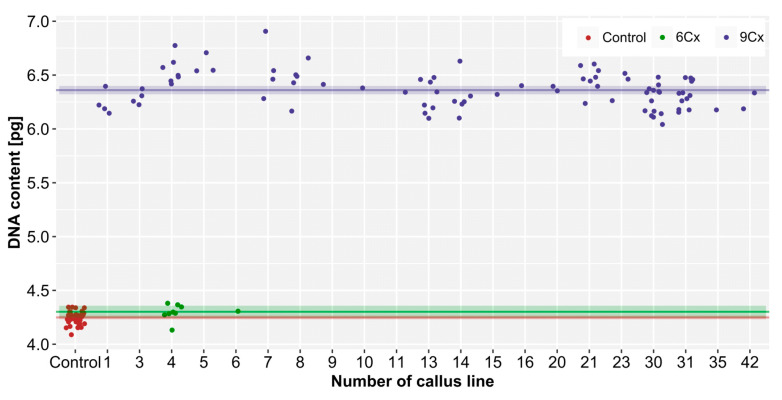
Nuclear DNA content of seedlings (6Cx control) and endosperm-derived plants (6Cx and 9Cx regenerants) of *Actinidia chinensis* var. *deliciosa*. The points represent 2C values of individual plants, and are scattered randomly to the right and left above the given number of callus lines to avoid overlapping the same or very similar values. The horizontal lines accompanied by semi-transparent bands in the appropriate colours show the mean values and 95% confidence intervals (CI) for the sets of measurements in order of DNA content. Note that from endosperm-derived callus line no. 4, both 6Cx and 9Cx plants were regenerated.

The 2C nuclear DNA content in control hexaploid plants ranged from 4.09 to 4.35 pg, with a mean value of 4.25 pg/2C (Figure 2); thus, the Cx-value was about 0.71 pg. The regenerated plants possessed a 2C DNA content between 4.14 and 6.91 pg. Nine regenerants, which had 4.14–4.38 pg 2C DNA, were considered to be hexaploids. The remaining 89 regenerated plants had an average genome size of 6.36 pg, which suggested nonaploidy. The standard deviations for the 6Cx control plants and 6Cx regenerants were almost the same (0.06 and 0.07; about 1.6% of the DNA content), but were higher for the 9Cx regenerants (0.19; about 3% of the DNA content). This indicated a higher degree of variation in the DNA content in the 9Cx regenerants. Welch’s test revealed statistically significant (*p* < 0.05) differences in the nuclear DNA content between 6Cx and 9Cx plants; there was no difference between 6Cx control plants and 6Cx regenerants.

### 2.3. Sex-Linked Molecular Markers Useful in Sex Identification of Endosperm-derived Callus Lines and Regenerated Plants

**Figure 3:**
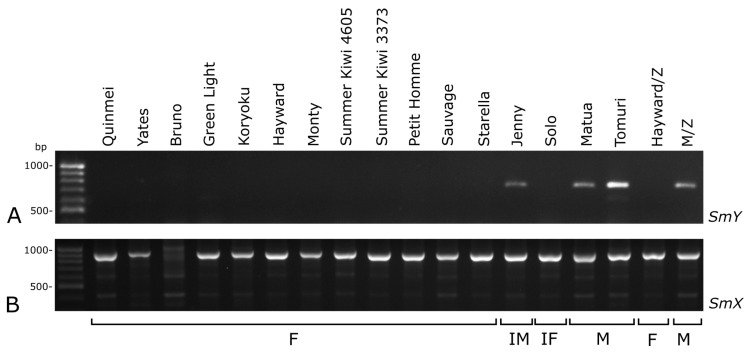
Amplification bands of *SmY* (**A**) and *SmX* (**B**) in selected cultivars of *Actinidia chinensis* var. *deliciosa*. Hayward/Z and M/Z indicate cv. Hayward and a male pollinator obtained from Zespri International Ltd. F—female, IF—inconsistent female, IM—inconsistent male, M—male.

Using selected sex-linked molecular markers on the known cultivars with different sexes, and the samples from the Zespri International Ltd., showed that *SmY* and both *SyGl* markers are suitable for determining the sex in *A*. *chinensis* var. *deliciosa*. The primers SMYf1 and SMYr1 amplified ca. 770 bp products in all of the analysed male cultivars but none of the analysed female cultivars (Figure 3A).

**Figure 4:**
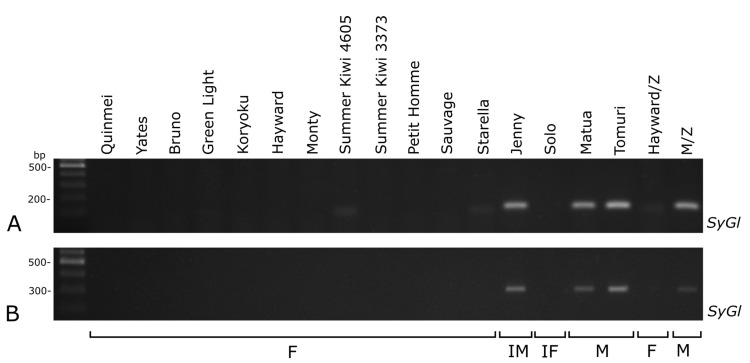
Amplification bands of the *SyGl* genic intron/exon (**A**) and *SyGl* 3′ promoter region (**B**) in selected cultivars of *Actinidia chinensis* var. *deliciosa*. Hayward/Z and M/Z indicate cv. Hayward and a male pollinator obtained from Zespri Internationatl Ltd. F—female, IF—inconsistent female, IM—inconsistent male, M—male.

The *SyGl* marker products (ca. 90 bp (*SyGl* genic intron/exon) and ca. 310 bp (*SyGI* 3’ promoter region)) were also present in all of the male samples but not in the female samples (Figure 4). For both of the markers (*SmY* and *SyGl*), the results in the two hermaphrodite cultivars were different. In the cultivar Jenny (inconstant male), the products of the markers were the same as in the male cultivars, while in the Solo cultivar (inconstant female), the products were absent (Figure 3A and Figure 4A). Application of the sex-specific RAPD markers in the conditions described by Shirkot et al. [21], and after our modifications did not result in an appropriate RAPD profile.

Because some male samples could be identified as female due to problems with DNA extraction or the PCR reaction, a positive control was required. Thus, the *SmX* sequence was selected, because it displays no correlation with the sex. Ca. 950 bp product was detected in all cultivars except ‘Bruno’, which produced some weaker bands (Figure 3B). 

**Figure 5:**
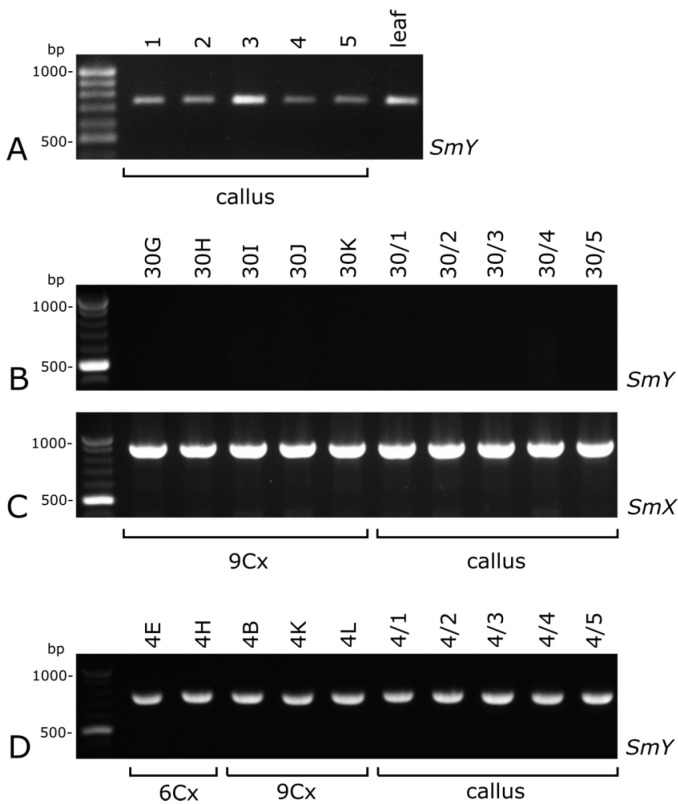
Amplification bands of *SmY* in leaf-derived callus from the male *Actinidia chinensis* var. *deliciosa* cv. Tomuri (**A**) endosperm-derived callus line number 30 (**B**) line number 4 (**D**) and plants regenerated from their respective calli (**B**,**D**). No *SmY* amplified bands indicates a female characteristic of a callus and 9Cx regenerants (**B**). Male characteristic of callus (**A**), 6Cx and 9Cx regenerants (**D**). Amplified bands of *SmX* as positive control (**C**).

To confirm that the marker *SmY* can be detected also in callus, this kind of tissue obtained from the leaf of the male cultivar ‘Tomuri’ was tested. The PCR product of *SmY* was present both in leaf and leaf-derived callus (Figure 5A).

Based on these results, for the further analysis of the sex of endosperm-derived callus the *SmY* marker was selected. When no products of this marker were obtained, an additional amplification of *SmX* was performed as the positive control. In 11 lines of endosperm-derived calli, the PCR product of SmY was detected (therefore, male callus), and in 10 lines, this PCR product was not found (female callus). The determination of sex for all 97 regenerants was consistent with the sex established for the each of the calli lines (Figure 5B–D, Table 1). Plants regenerated from a particular male line of callus tissue could differ in their DNA content (6Cx or 9Cx) even though their sex was the same (Figure 5D). Thus, *SmY* was detected at all stages of culture (from callus to regenerants), regardless of the level of ploidy.

## 3. Discussion

### 3.1. Callus Multiplication, Plants Regeneration, and Acclimatization to Ex Vitro Condition

The present study is a continuation of the report about organogenesis from endosperm-derived callus in kiwifruit [15]. In this study, the percentage of shoot regeneration was similar to that in the previous report [15] on the medium with 0.5 mg/l TDZ. Some callus lines have been multiplied and maintained the ability of shoot bud regeneration for over four years [27]. The efficiency of acclimatization was rather high (nearly 79%). Similar studies on *A. chinensis* reported a noticeable higher efficiency; however, the cultivar used had a lower ploidy level than ‘Hayward’ [14]. Generally, the efficiency of acclimatization of endosperm-derived plants varies in different species, e.g., in *Morus alba* was slightly lower (71%) [28], in *Carica papaya* was similar (78%) [29], and in *Melia azedazach* higher (100%) [30] than descripted in this paper.

### 3.2. Nuclear DNA Content and Ploidy of the Regenerants

The nuclear DNA content of hexaploid *A*. *chinensis* var. *deliciosa* (4.25 pg/2C; control seedlings) was similar to that previously reported (4.4–4.5 pg/2C; [6]). Our data on the 2C DNA content of control hexaploid plants enabled us to establish the Cx-value (about 0.71 pg), and, in turn, the DNA ploidy of plants regenerated from endosperm tissue culture, which were expected to possess 9Cx DNA (about 6.39 pg). The mean value of 6.36 ± 0.19 pg confirmed the nonaploid status of 90% of the regenerants. Earlier studies claimed that plants regenerated from endosperm tissue directly, or indirectly via a callus, contain in their somatic cells amounts of nuclear DNA that are characteristic of endosperm tissue [3,4,15]. However, in this study, some regenerants contained a DNA content that deviated from the mean value by approx. 10% (genome size range 5.57–6.91 pg), which suggests somaclonal variation occurred during in vitro culture. This phenomenon is common in plant material cultured in vitro, especially when regenerated via callus [31]. Variations in chromosome number (aneuploidy) might be the reason for our observed variation in the amounts of nuclear DNA. In a previous studies [15], atypical leaves of endosperm-derived shoots were indicative of aneuploidy. The appearance of regenerants with a DNA content similar to the mother plant (hexaploid) might be due to the introduction of some mother plant or embryo cells as contaminants in the endosperm during isolation from the seed. This has been noted also, for example, during chromosome counting in cultured cell aggregates derived from endosperm tissue of *Gomortega keule*, where some cells were found to possess a diploid number of chromosomes [32]. 

### 3.3. Sex-Linked Molecular Markers Identify Sex of In Vitro and Ex Vitro Samples

We attempted to obtain RAPD markers, described by Shirkot et al. [21] as male- and female-specific, but the products of their primers were not sex-specific, probably because of the low repeatability of RAPD method [33]. Use of other sex-linked molecular markers (*SmY*, *SyGl* genic intron/exon, and *SyGl* 3′ promoter region) confirmed their usefulness in determining sex in *A*. *deliciosa*, and it was also possible to recognize self-fertile individuals, such as inconsistent males and females, probably because hermaphroditism is caused by additional genes in this species [16]. Kiwifruit has an active-Y sex determination system [18], and all Y-specific markers correctly identified the sex of the male and female cultivars, especially the plants from Zespri International Ltd., the source material for the in vitro cultures. Similar analyses with the *SyGl* marker on tetraploid and hexaploid plants of *A*. *arguta* confirmed that the product of this gene is connected with the male sex, but not with the ploidy variant [23]. These results suggest that the molecular markers of the Y chromosome reveal the sex of the analyzed samples from in vitro cultures regardless of their ploidy level. The regenerants that were obtained from endosperm-derived callus were usually nonaploids with (X)_7_ XX for females and (X)_7_ XY for males, according to the polyploid description proposed by McNeilage [16]. This means that in the nonaploids, the concentration of template DNA from Y chromosomes is relatively lower than in the hexaploid male plant, and therefore detection of the Y chromosome-connected markers may be prevented. Our results reveal that the *SmY* marker can be used successfully to detect the Y chromosome in calli and regenerants. In male hexaploid plants, there is one Y chromosome per five X chromosomes and six sets of autosomes, but in nonaploids, there is also one Y-chromosome but per eight X chromosome and autosome sets. Therefore, our analyses are sufficiently sensitive to identify male plants, even nonaploids. Moreover, these markers can be useful for identifying the sex at even the calli stage. Thus, this method can be utilized in kiwifruit breeding, enabling early selection of callus of the desired sex, and consequently that of callus-derived regenerants. Sex identification based on Y-specific markers may be biased, however, by mutations such as deletions or translocations of a marker-containing fragment of chromosome Y [34]. It is also possible that two Y chromosomes are present because of fertilization by gametes with additional Y-chromosomes due to meiotic aberrations [12]. While using the marker-based method such cases cannot be identified, the aim of plant selection usually is to obtain female plants that do not harbor a Y chromosome, so in this respect it is irrelevant as to whether the males contain one or more.

## 4. Materials and Methods

### 4.1. Culture of Endosperm-Derived Callus, Plant Regeneration and Acclimation to Ex Vitro Conditions

The study was performed on material derived from commercially available fruits of *A*. *chinensis* var. *deliciosa* ‘Hayward’ (Zespri International Limited). Sterilization of fruits, acquisition of seeds, seed coat removal, and the dissection of embryo and endosperm were performed according to protocols described previously [15,35]. For organogenic callus development and shoot bud induction, isolated endosperms were placed onto organogenic callus induction medium (OCIM) in 60 mm diam Petri dishes, MS medium [36], that contains salts and vitamins (Duchefa), with 30 g/l sucrose, 6 g/l Plant Agar (Duchefa) supplemented with 0.5 mg/l thidiazuron (Sigma). The dishes were sealed with Parafilm and incubated at 25 °C in the dark. The callus line was obtained by dividing proliferating calli into clumps, which continued to grow. Each callus line obtained from one particular endosperm explant was numbered individually. After the formation of shoot buds, cultures were transferred to 16-h photoperiods under cool-white fluorescent tubes (90 µmol photons m^−2^s^−1^). The plants that regenerated were transferred from the OCIM onto half-strength MS medium in culture containers, Magenta vessels (Sigma), for the root development. Regenerants from a particular callus line, in addition to the previously assigned number, were also marked with a code letter. Because kiwifruit needs as many as four to eight years to form flowers [25], no blooming plants were obtained. 

Plants developed from seeds were used as controls. Seeds were scarified with a lancet and placed on half-strength MS medium supplemented with 20 g/l sucrose and 6 g/l Plant Agar (Duchefa). The germinated seedlings were transferred into Magenta vessels (Sigma) and incubated in the photoperiod conditions above. The seedlings and endosperm-derived regenerants with well-developed roots (Figure 4A) were gently washed, transferred into pots with a commercial substrate for seeding (Substral), and wrapped with plastic bags. During the next few days, the bags were perforated and then completely removed after one week. The plants were maintained at the temperature and photoperiod conditions above.

### 4.2. Flow Cytometric Analyses

Ninety-seven plants that were regenerated from an endosperm-derived callus were selected on the basis of leaves that were non-vitrified and with a shape typical for the taxon. Forty-one seedlings were used as controls. The leaf samples were prepared and analyzed as previously described [37], using propidium iodide (PI) for DNA staining. *Petunia hybrida* P×Pc6 (2.85 pg/2C; [38]) served as the internal standard. Nuclear DNA content was estimated using a CyFlow SL Green (Partec GmbH, Münster, Germany) flow cytometer, equipped with a high-grade solid-state laser with green light emission at 532 nm, long-pass filter RG 590 E, DM 560 A as well as with side (SSC) and forward (FSC) scatters. For each sample, nuclear DNA content in 5000–8000 nuclei was measured using linear amplification. Histograms were evaluated using a FlowMax program (Partec GmbH, Münster, Germany). The coefficient of variation (CV) of the G_0_/G_1_ peak of the *Actinidia* species ranged from 2.72% to 6.64%. The nuclear DNA content was calculated on a histogram of fluorescence intensities using the linear relationship between the ratio of the 2C peak positions of *Actinidia/Petunia*.

Cx-value was calculated on the basis of the genome size of control hexaploid plants and applied to establish the DNA ploidy of regenerants. Cx refers to the DNA content of a monoploid genome with chromosome base number x [39]. Thus, the abbreviation 6Cx is used for a 2C DNA content of a hexaploid plant, and 9Cx of a nonaploid; the latter originated from endosperm cells with 3C DNA content.

### 4.3. Sex-Linked Molecular Markers 

The vines of 16 cultivars (Table 2) from the ex vitro conditions (pots with soil or commercial orchard) were the source of leaves for the test to determine the most appropriate sex-linked molecular markers. Samples were obtained from a private collection (owner Christian De Kezel, Sint Amandsberg, Belgium), the Palm Center of Bulgaria (Botanical Garden, Plovdiv) and from a commercial orchard of Zespri International Ltd. (Cisterna di Latina, Italy). The samples of male pollinators that were acquired from the same company were marked as M (Table 2).

For leaf-derived callus, the leaves of the male cv. Tomuri were sterilized, cut into 1 cm squares, placed on a non-organogenic callus-induction medium (NCIM) supplemented with 2 mg/l 2,4-D (Sigma) and 5 mg/l kinetin (Sigma) and incubated at 25 °C in the dark. Five independent samples from each callus line (leaf-derived callus and 21 lines of endosperm-derived callus) were collected. One to three samples were also taken from cultivars and regenerated plants. A total of 97 regenerants were analyzed, from one to fifteen plants per callus line (Table 1).

DNA was extracted according to Gawel and Jarret [40]. Sex-specific markers were chosen as recommended especially for kiwifruit, formerly described as *A*. *chinensis* and *A*. *deliciosa* [20,21,23]. The male-specific markers were amplified using three pairs of PCR primers: SMYf1 and SMYr1 (SmY) [20]; SyGl-spe-prom2F and SyGl-prom-end-indelR (SyGl 3′ promoter region); SyGl-selectF and SyGl-selectR (SyGl genic intron/exon) [23] (Table 3). Another pair of primers (SMXf and SMXr [20]) was used as the positive control to exclude any false sex identification due to, for example, the poor quality of the template DNA. The PCR conditions were the same for all of markers. The reaction mixture (15 μl total volume) contained a 1x concentration of DreamTaq buffer (Thermo Scientific), 0.25 mM dNTPs (Thermo Scientific), 0.25 μM of each primer, 1.13 U DreamTaq DNA Polymerase (Thermo Scientific), and 50 ng DNA template. Amplification was performed using the following program: an initial denaturation step at 94 °C for 5 min., 35 cycles consisting of a denaturation step at 94°C for 45 s, a primer annealing step at 57 °C for 45 s, a primer extending step at 72 °C for 2 min, and a final extending step at 72 °C for 10 min. The other tested markers were RAPD primers, described as male-specific (OPC-05, OPN-01) and female-specific (OPA-01, OPA-02, OPA-8, OPA-11, OPA-16, and OPB-01) [21]. In this case, the reaction was performed in a total volume of 10 μl and contained a 1x concentration of DreamTaq buffer (Thermo Scientific), 0.25 mM dNTPs (Thermo Scientific), 1 μM primer, 0.5 U DreamTaq DNA Polymerase (Thermo Scientific), and 10 ng DNA template. The thermocycler program consisted of an initial denaturation at 94 °C for 1 min, followed by 40 cycles of 30 s at 93 °C, 1 min at 34 °C, 2.5 min at 68 °C, and a final extending step at 68 °C for 5 min. The PCR and RAPD products were separated in 1–1.5% agarose gels stained with SimplySafe (Eurx).

### 4.4. Statistical Analyses

Statistical analyses were performed using R software [41]. Three groups of samples (6Cx control, 6Cx and 9Cx regenerants) were compared by DNA content [pg]. For all data groups, the mean, confidence intervals for 95% (CI) and standard deviation values were calculated using appropriate functions (*mean()*, *CI()*, *sd()*, respectively). For each comparison, the equalities of the variances between the groups were tested using Levene’s test of equality of variance (*leveneTest()* function). Because variances were not homogenous (*p* < 0.05), Welch’s heteroscedastic F test (*welch.test()*) was used to compare the data between the groups. The difference was regarded as significant when *p* < 0.05. The ggplot2 package [42] was used to generate Figure 2.

## 5. Conclusions

We have obtained rooted and acclimatized to ex vitro conditions nonaploid *A*. *chinensis* var. *deliciosa* plants from endosperm-derived callus. The majority of regenerated plants possess a 9Cx DNA content, which confirms their endosperm origin and nonaploid status (2n = 9x). However, the presence of hexaploid regenerants emphasizes the need for ploidy determination of all endosperm-derived plants. Because the Y chromosome is regarded as the one that determines the male sex, the sex-linked marker *SmY* can be used as a reliable marker for this chromosome regardless of the type of sample or its ploidy. Consequently, using this marker, determination of the sex of a callus is a useful predictor of male- or femaleness before plant regeneration. This method can be useful for sex selection in breeding programs of *A*. *chinensis* var. *deliciosa*, especially when the sex of the source plants for callus production (and subsequent explants) is unknown. Our results may be important for breeders, offering them the efficient early selection method of desired female lines, especially because presented-here protocols were established for the most popular kiwifruit cv. Hayward. Obviously, the futures studies on, e.g., fertility and possible meiosis disturbances in regenerated plants are needed.

## Figures and Tables

**Table 1:** Twenty one endosperm-derived calli lines and 97 regenerated plants of *Actinidia chinensis* var. *deliciosa* analyzed with sex-linked molecular markers *SmX* and *SmY*.

**Female**												
**Callus ID**	1	6	9	10	14	20	21	23	30	35		**10 lines**
**Number** **of Regenerants**	4	1	1	1	6	2	8	3	14	1		**Total Number: 41**
**Male**												
**Callus ID**	3	4	5	7	8	11	13	15	16	31	42	**11 lines**
**Number** **of Regenerants**	4	15	3	4	5	1	8	1	1	12	2	**Total Number: 56**

**Table 2:** Cultivars of *Actinidia chinensis* var. *deliciosa* used in sex-linked molecular markers tests.

Sex	Cultivar		Source
**Female**	‘Hayward’		Private collection (Belgium) and Zespri International Ltd. (Italy)
	‘Bruno’ ‘Koryoku’ ‘Petit Homme’ ‘Sauvage’ ‘Summer Kiwi 3373’‘Yates’	‘Green Light’ ‘Monty’ ‘Quinmei’ ‘Starella’ ‘Summer Kiwi 4605’	Private collection (Belgium)
**Male**	M *		Zespri International Ltd. (Italy)
	‘Matua’		Private collection (Belgium)
	‘Tomuri’		Palm Center (Bulgaria)
**Inconsistent Female**	‘Solo’		Private collection (Belgium)
**Inconsistent Male**	‘Jenny’		Private collection (Belgium)

* Male pollinator.

**Table 3:** Sex-linked molecular markers.

Marker		Sex-Specificity	References
*SmY*		male	[20]
OPC-05	OPN-01	male	[21]
OPA-01 OPA-02 OPA-8	OPA-11 OPA-16 OPB-01	female	
*SyGl* genic intron/exon		male	[23]
*SyGl* 3′ promoter region			

## Data Availability

The datasets generated during and/or analyzed during the current study are available from the corresponding author on reasonable request.
